# Lipiodol retention pattern after TACE for HCC is a predictor for local progression in lesions with complete response

**DOI:** 10.1186/s40644-019-0260-2

**Published:** 2019-11-15

**Authors:** Marco Dioguardi Burgio, Riccardo Sartoris, Claudia Libotean, Magaly Zappa, Annie Sibert, Valérie Vilgrain, Maxime Ronot

**Affiliations:** 1 Department of Radiology, APHP, University Hospitals Paris Nord Val de Seine, Beaujon, Clichy, Hauts-de-Seine, 100 Boulevard du Général Leclerc, 92118 Clichy, France; 2 University Paris Diderot, Sorbonne Paris Cité, Paris, France; 3 INSERM U1149, CRI, Paris, France

**Keywords:** Carcinoma, hepatocellular, Ethiodized oil, Chemoembolization, therapeutic

## Abstract

**Background:**

To evaluate the predictive value of the lipiodol retention pattern for local progression of HCC with a complete response (CR) on CT according to mRECIST criteria after a first session of conventional chemoembolization (cTACE).

**Methods:**

From January 2014 to May 2016 all consecutive patients undergoing a first cTACE session for HCC were identified. Inclusion criteria were the presence of ≤3 HCCs and available pre- and post-cTACE CT. Tumor response was classified according to mRECIST criteria. The analysis focused on tumors with a CR. The lipiodol retention pattern in these tumors was classified as complete (C-Lip, covering the entire tumor volume), or incomplete (I-Lip). Local progression was defined as the reappearance of areas of enhancement on arterial-phase images with washout on portal/delayed phase images within 2 cm from treated tumors on follow-up CT.

**Results:**

The final population included 50 patients with 82 HCCs. A total of 46 (56%) HCCs were classified with a CR, including 16 (35%) with I-Lip, and 30 (65%) with C-Lip. After a median follow-up of 14 months (3.2–35.9 months), 15/16 (94%) and 10/30 (30%) of I-Lip and C-Lip HCCs showed local progression on CT, respectively (*p* < 0.001), with no significant difference in the time to progression (mean 11.1 ± 2 vs. 13.4 ± 3 months for I-Lip and C-Lip, respectively *p* = 0.51).

**Conclusions:**

HCCs with incomplete lipiodol retention after a first cTACE session have a high risk of local progression even when there is a CR according to mRECIST, and should be considered to be incompletely treated.

## Background

Transarterial chemoembolization (TACE) is the reference treatment in patients with intermediate HCC that are not eligible for curative treatment such as ablation, surgery or liver transplantation [1]. An objective response after treatment has been identified as an independent prognostic factor [2, 3]. TACE is also the most common bridge therapy in patients waiting for liver transplantation, because it has been shown to improve patient drop out [4, 5]. Moreover, response to TACE, and especially a complete response (CR), has been shown to be associated with a better outcome after transplantation, and authors have suggested that this should be used as a selection criterion in patients waiting for liver transplantation [6].

RECIST 1.1 are the most commonly used criteria to evaluate tumor response after oncological treatment [7]. However, RECIST 1.1 criteria are not accurate in HCC after locoregional therapy such as TACE, as they have been shown to largely underestimate tumor necrosis [8]. For this reason, a modified version (mRECIST) has been proposed by the American Association for the Study of Liver Diseases [9], which takes into account the single axial measurement of the hypervascular portion of the tumor considered to represent viable tissue. Thus, after treatment, a tumor with no hypervascular component is considered to be necrotic and according to mRECIST criteria, a complete response (CR) is achieved when all hypervascular components have disappeared. mRECIST has been shown to be reproducible for the differentiation of a response and non-response after TACE [10], and to improve identification of complete or almost complete tumor necrosis [11].

However, several studies have suggested that mRECIST may overestimate tumor response compared to a pathological assessment of necrosis [10, 11]. In conventional TACE (cTACE), which uses an emulsion of antimitotic drugs and lipiodol, this may occur because hyperattenuation on CT from the high iodine concentration of lipiodol may mask underlying hyperenhancement of portions of the tumor [12]. Thus, certain authors recommend using MR imaging to assess tumor response [12]. Nevertheless, many institutions, especially those that perform high-volume cTACE, partially or exclusively monitor patients with CT. These teams believe that the lipiodol retention pattern is not only an issue, but is also informative. Indeed, Takayasu et al. have shown that when lipiodol retention is considered to be a necrotic area there is a good correlation with pathological necrosis [13]. Moreover, Dioguardi Burgio et al. recently demonstrated that in nodules showing a CR according to mRECIST, those with complete lipiodol retention had a significantly higher amount of necrosis than those with a CR but with incomplete lipiodol retention [11].

As a result, we hypothesized that the outcome of HCCs with a CR according to mRECIST after a cTACE session may differ according to the pattern of complete or incomplete lipiodol retention.

The aim of this study was to investigate the predictive value of the lipiodol retention pattern after one session of cTACE for the local progression of HCC.

## Materials and methods

### Patients selection

This single center retrospective study was performed in BLINDED and approved by the institutional review board. Informed consent was waived because of the retrospective design. From January 2014 to May 2016 treatment-naïve patients who underwent a first session of cTACE for the treatment of HCC were retrieved from the medical database of our institution. Inclusion criteria were (i) the presence of at least one HCC according to EASL clinical practice guidelines [1], (ii) contrast-enhanced computed tomography (CT) before the cTACE procedure, and during follow-up and (iii) the presence of up to three HCCs to better identify local progression of individual tumors. Tumors that could not be evaluated for response according to mRECIST criteria, (i.e. hypoenhanced on arterial phase and/or infiltrative tumors) were not included in the study.

A total of 86 patients with cirrhosis underwent a first session of cTACE during the study period. Thirty-six patients were excluded for the following reasons: a) more than 3 HCCs, (*n* = 10); b) systemic treatment before TACE (*n* = 3: two patients receiving sorafenib and one patients receiving gemcitabine and oxaliplatin); and c) lack of follow-up CT images (*n* = 11); infiltrative or hypovascular HCC (*n* = 12).

**Fig. 1:**
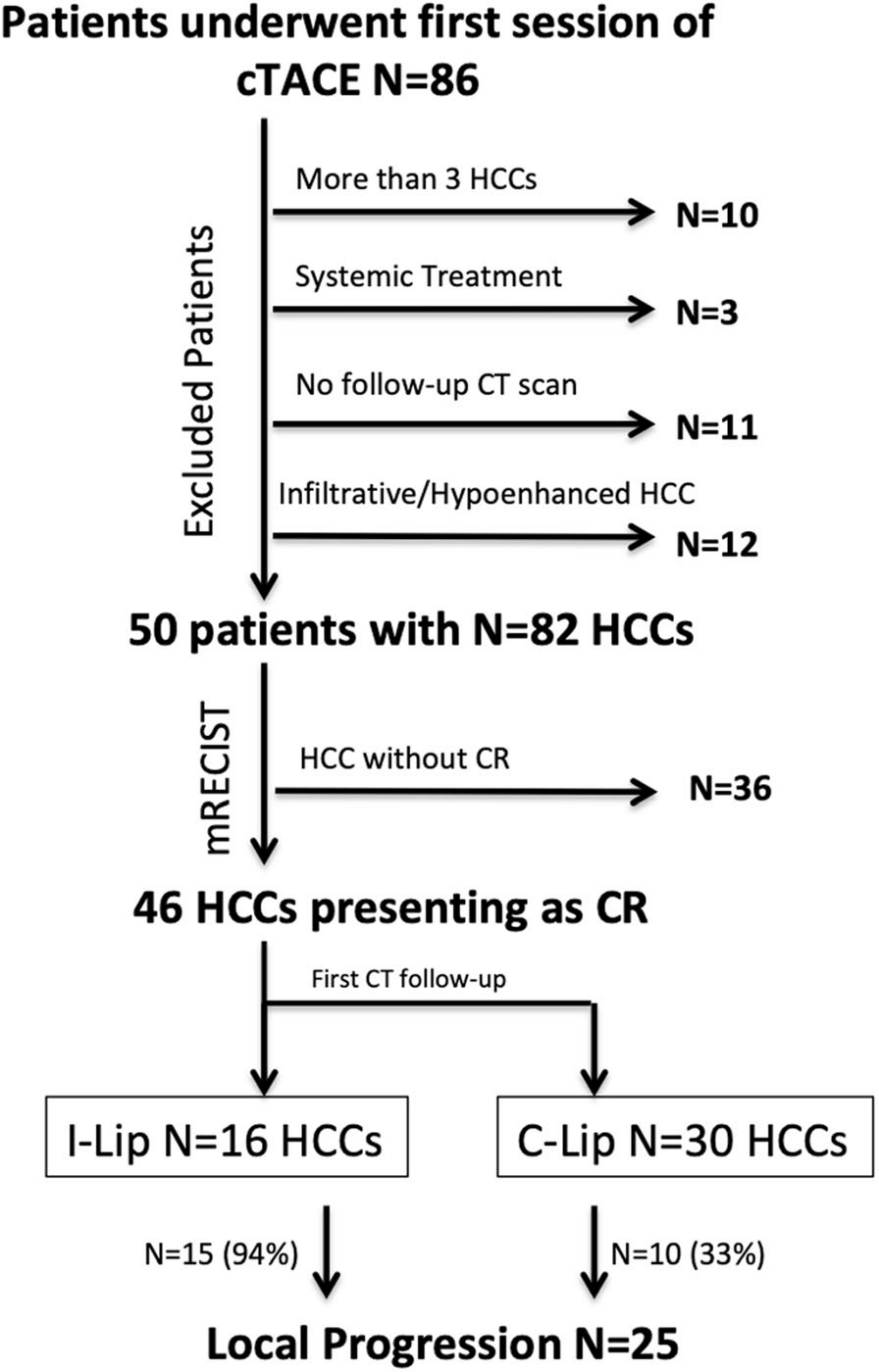
Study design. cTACE: conventional Transarterial Chemoembolization. C-Lip: Complete lipiodol retention, HCC: Hepatocellular Carcinoma. I-Lip: Incomplete lipiodol retention, mRECIST: Modified Response Evaluation Criteria in Solid Tumors

The final population of this study included 50 patients (mean age 62 ± 12 yo; 45 men [90%]) with 82 HCCs (mean 27 ± 15 mm, (10–75 mm)). Figure 1 shows the patient flow chart.

### Conventional TACE procedure

The decision to perform TACE was discussed by a tumor board for each patient (including oncologists, hepatologists, radiologists, interventional radiologists and hepatic surgeons). Before each cTACE session a team of interventional radiologists chose the most appropriate approach for each patient. All cTACE procedures were performed according to recent expert consensus guidelines [14].

All cTACE procedures were performed under local anesthesia by one senior radiologist (with at least 5 years of experience in interventional oncology of the liver). A right femoral approach was obtained with Seldinger technique using a 5F introducer sheath. Digital subtraction angiography or 3-D cone beam CT angiography was used to identify each target lesion and vascular feeders. The celiac trunk or superior mesenteric artery was catheterized using a 5F Cobra or Simmons angiographic catheter. Treatment was performed as selectively as possible with 2.4–2.7-F microcatheters and involved the injection of a mixture of chemotherapy (up to 60 mg of doxorubicin; Adriamycin; Pharmacia Upjohn,) and emulsified poppy seed oil (Lipiodol, Gerbet). Associated embolization was performed by injection of gelatin sponge (Gelitaspon, Gelita Medical B.V.) or polyvinyl alcohol particles (Bead Block, Biocompatibles). cTACE was considered to be selective if the treatment was injected directly into tumor feeders, and as nonselective otherwise.

### CT protocol

All pre and post-cTACE contrast-enhanced abdominal CT were performed with a multidetector CT (64-detector LightSpeed VCT; GE Healthcare,). A multiphase acquisition was obtained as follows: after an unenhanced abdominal scan, a nonionic iodinated contrast agent containing 350 mg I mL–1 was administered intravenously through a 16–18 gauge cannula. A mean 2 mL/kg of contrast medium was injected via an antecubital vein at 4 mL/s. No oral contrast medium was used. Arterial, portal, and late venous phase acquisitions were obtained at 35, 80, and 180 s, respectively, following contrast injection. A maximum thickness per slice of 2.5 mm was obtained for each acquisition.

### Image analysis and tumor response

#### Baseline tumor characteristics

Pre- and post-cTACE contrast-enhanced CT images were reviewed by two senior abdominal radiologists (XX and YY with XX and YY years of experience in the field of liver imaging). Images were reviewed in consensus on a picture archiving and communication system (PACS) (Carestream Health). The following items were evaluated and recorded for each treated tumor: a) largest diameter (in mm); b) the presence of a capsule defined as a smooth, uniform, sharp border around the lesion that was clearly thicker or more conspicuous than fibrotic tissue around the background nodules, and visible as an enhancing rim in portal or delayed phase acquisitions; c) the presence of bland portal vein thrombosis; and d) location according to Couinaud’s liver segment classification.

#### Lipdiodol retention pattern on first follow-up CT

**Fig. 2:**
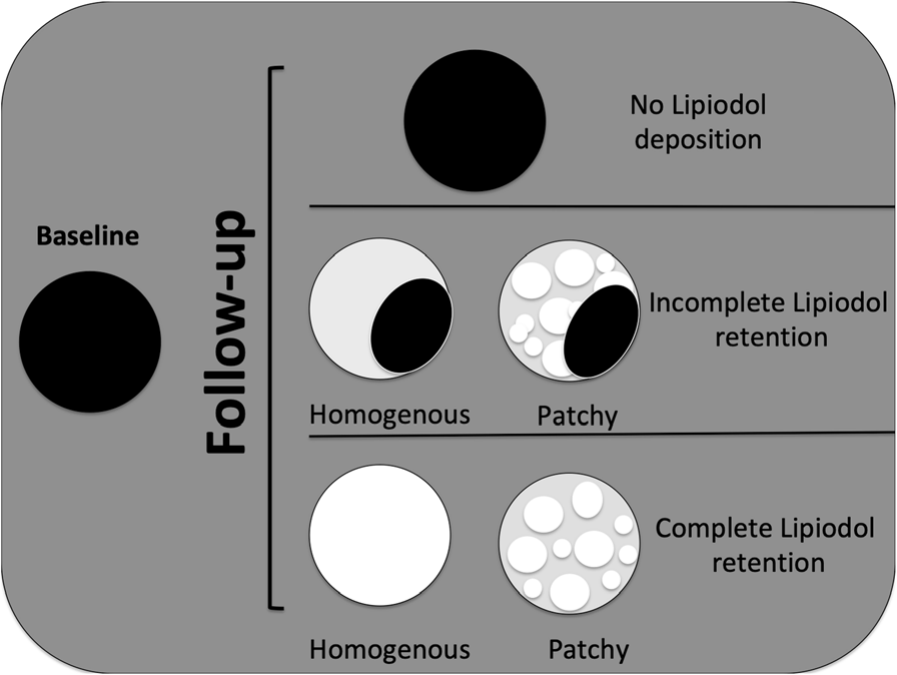
Schematic representation of lipiodol retention patterns on precontrast CT after cTACE

The lipiodol retention pattern was assessed on pre-contrast images of post-TACE CT performed 4 to 6 weeks after treatment (mean 38 ± 9 days; (20–73 days)). To ensure that the entire tumor volume was explored, images were compared with pre-cTACE hepatic arterial phase CT. Lipiodol retention was considered to be complete if the entire nodule volume was hyperattenuating compared to the surrounding liver parenchyma on pre-contrast images (retention could be either homogenous or patchy as long as the whole tumor volume showed lipiodol retention). Lipiodol retention was considered to be incomplete if the nodule volume showed only partial hyperattenuation on post-TACE pre-contrast images. Figure 2 provides schematic examples of the two retention patterns.

#### Tumor response and local progression

Tumor response was evaluated in a tumor-by-tumor analysis. Tumors were individually classified according to mRECIST [9] as a complete response (CR), partial response (PR), stable disease (SD) or progressive disease (PD). The current study focused on nodules showing a CR, i.e. with no hyperenhanced portions on arterial phase images after cTACE. Therefore, patients without a CR in tumors on first follow-up CT were excluded from the local progression analysis, because a second cTACE session was required [15].

**Fig. 3:**
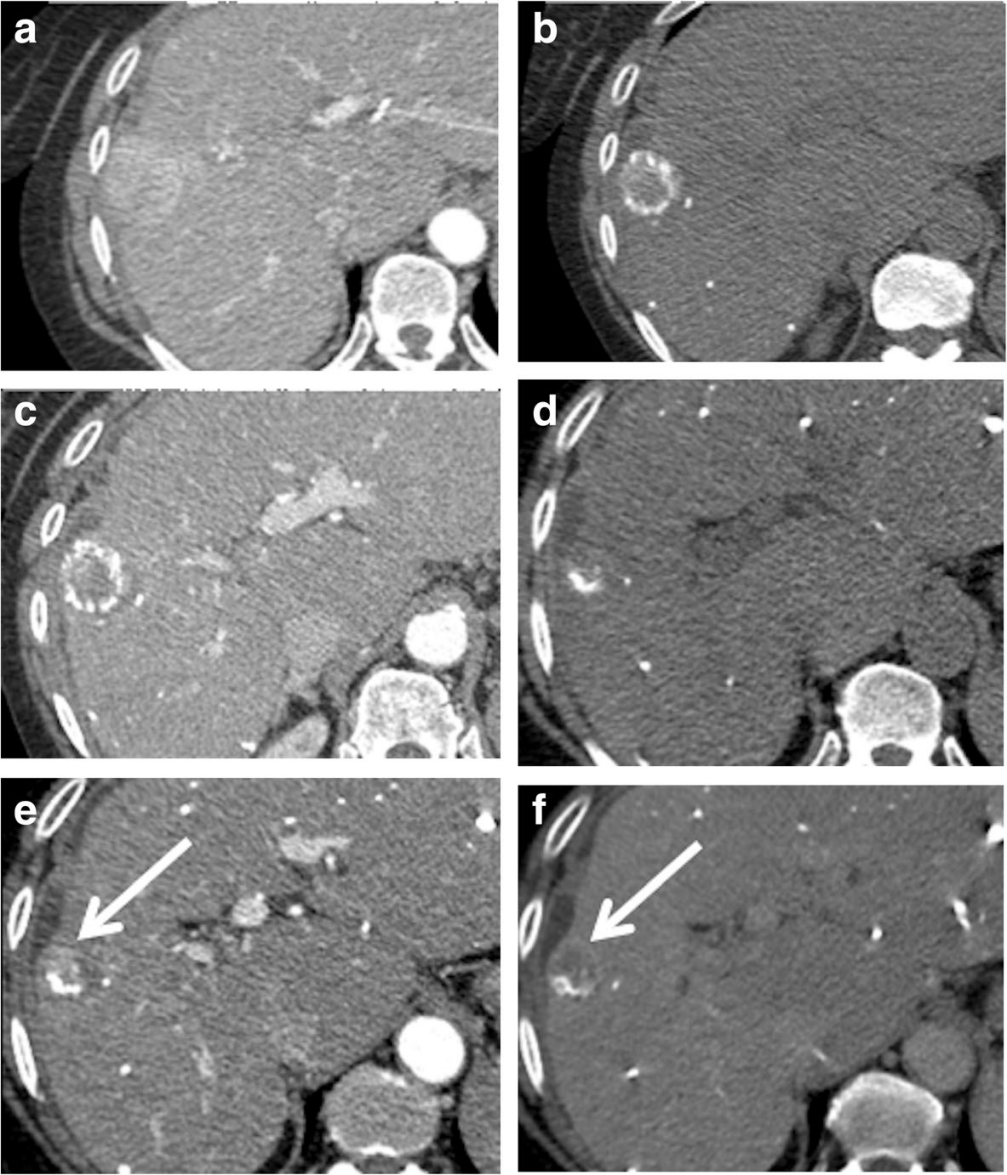
Local progression of an HCC showing a complete response according to mRECIST with incomplete lipiodol retention in a 59 yr-old female with HCV related cirrhosis. **a** baseline CT image obtained during hepatic arterial phase (HAP) shows a hyperehanced HCC in the right liver lobe. **b** and **c** first follow-up CT images showing the lesion presenting with incomplete lipiodol retention on a precontrast image (**b**), while images obtained during HAP (**c**) show no residual enhancement of the lesion. Millimetric lipiodol depositions were depicted in the right liver, suggesting the presence of pre-existing yet undetected small tumor foci. This CT showed the appearance of a lesion in the left liver lobe (not shown in the figure), for which the patient underwent a second TACE session targeting only the left liver lobe. **d**-**f** 3 months follow-up CT images obtained during precontrast (**d**), HAP (**e**) and delayed (3′) phase (**f**) show the reappearance of a hyperenhancing nodular portion on HAP (**e**) with washout on delayed (3′) phase (**f**) consistent with local progression (arrow)

**Fig. 4:**
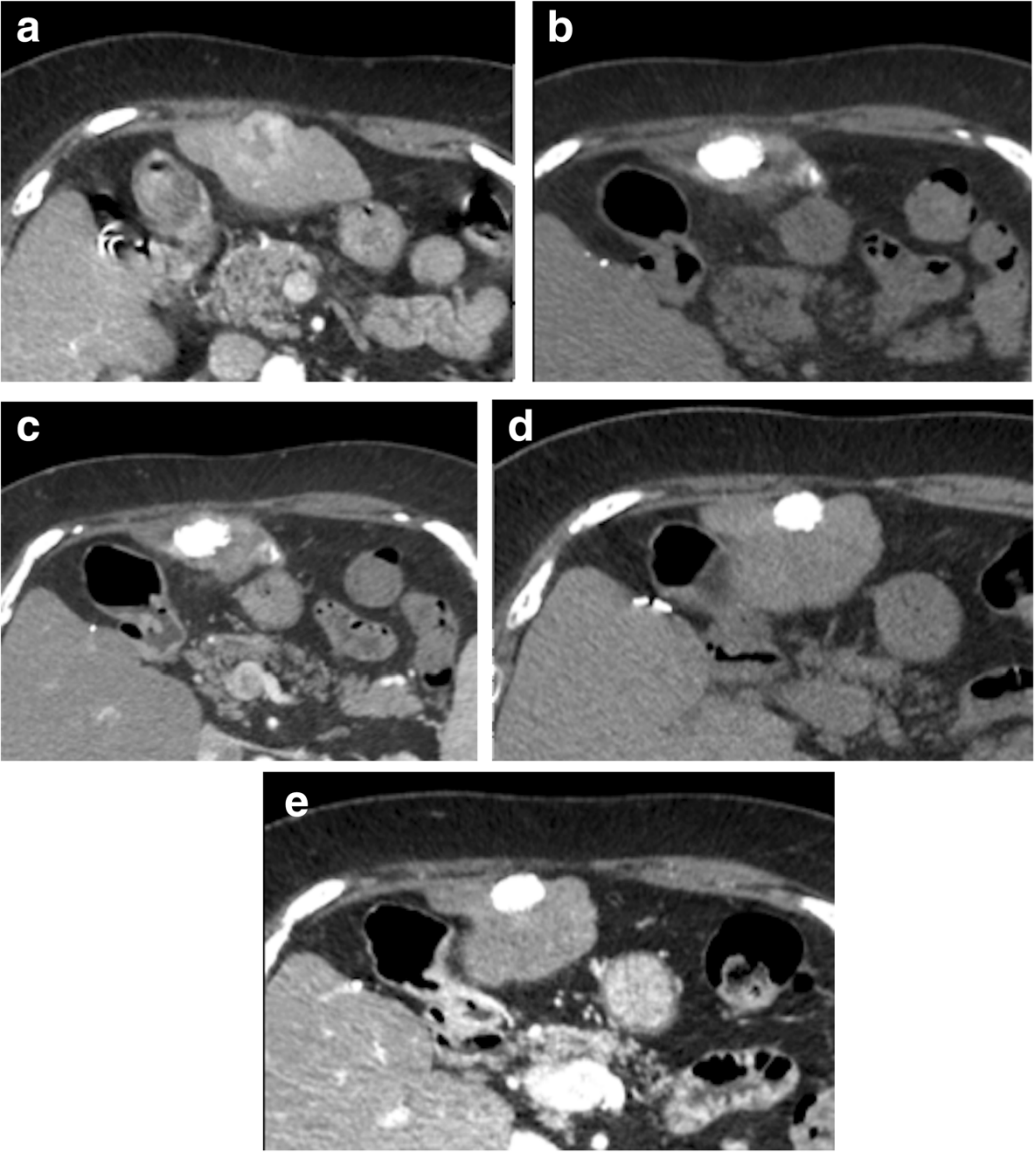
Absence of local progression of an HCC showing a complete response according to mRECIST with complete lipiodol retention in an 83 yr-old male with HBV related cirrhosis. **a** baseline CT image obtained during hepatic arterial phase (HAP) shows a hyperenhancing HCC in the left liver lobe. **b** and **c** first follow-up CT images showing the lesion presenting with complete lipiodol retention on the precontrast image (**b**) and no residual enhancement on HAP (**c**). **d**-**e** 24 months follow-up CT images obtained during unenhanced (**d**) and HAP (**e**) show that the nodule has decreased in size with persistent complete lipiodol retention in the lesion. No enhancing lesion in noticed on HAP (**e**)

Each tumor was followed until local progression occurred (follow-up CT was performed every 3 months in patients), which was defined as the appearance of a hyperenhanced nodular portion with washout on portal or delayed phase images (Fig. 3), within two cm from the treated lesion. In case of doubt of local progression, hepatic MR imaging was performed. The time from cTACE to local progression was recorded. If a tumor showed no local progression on the last available follow-up CT (at least 6 months after TACE), the nodule was considered to be a persisting CR (Fig. 4).

#### Statistical analysis

Data were expressed as means, standard deviations and ranges, or number of cases and frequencies, as appropriate. A Fisher exact test or a Chi-2 test was used to compare frequencies. The Student t test or Mann-Whitney test was used to compare continuous variables according to data distribution. The clinical, tumor and technical characteristics of the lipiodol retention pattern were identified by univariate analysis, and significant factors (*p* < 0.1) were entered into a binary logistic regression model. Association between lipiodol retention pattern and local progression was also weighted by means of odds ratio (OR). Time to local progression was defined as the time between the cTACE session and the first local progression. Factors associated with local progression were identified by a Cox-Mantel model, and provided with the corresponding hazard ratios. A *p* value < 0.05 was considered to be significant. All analyses were performed with the Statistical Package for the Social Sciences software, version 20.0 (SPSS Inc.)

## Results

### Patients and tumor baseline characteristics

Population and tumor characteristics are summarized in Table 1.

**Table 1:** Patient and tumor baseline characteristics

Population characteristics (*N* = 50)	
Gender M/F	45/5 (90/10%)
Mean age (years) ± SD (range)	62 ± 12 (20–83)
Causes of cirrhosis	
- Alcohol consumption	4 (8%)
- NAFLD	5 (10%)
- Alcohol + NAFLD	7 (14%)
- HBV	10 (20%)
- HVC	23 (46%)
- Other	1 (2%)
Number of HCC	
-1	23 (46%)
-2	22 (44%)
-3	5 (10%)
Mean HCC size (mm) ± SD (range)	27 ± 15 mm (10–75)
AFP (μg /L) ± SD (range)	299 ± 1356 (1.3–9395)
Serum bilirubin (μmol/L) ± SD (range)	16.57 ± 11 (3–54)
Serum albumin (g/L) ± SD (range)	36.5 ± 8.3 (21–57)

*AFP* Alpha-fetoprotein, *HBV* Hepatitis B virus, *HCC* hepatocellular carcinoma, *HCV* Hepatitis C virus, *NAFLD* Non-alcoholic fatty liver disease, *SD* standard deviation

All 50 patients had cirrhosis. The causes of cirrhosis were alcohol-related (*n* = 4), HCV (*n* = 23), HBV (*n* = 10), and other (*n* = 13). Before cTACE, patients had a mean 1.6 (1–3) HCCs, a mean 27 ± 15 mm (10 – 75 mm) in diameter. HCC was solitary in 23/50 patients (46%). Twenty-three lesions (28%) presented with a peripheral capsule appearance. Subsegmental portal vein thrombosis was observed in 2/50 (4%) of patients.

### cTACE characteristics

cTACE was selective in 61/82 (74%) nodules. Arterial-phase cone beam CT was used for treatment guidance in 27/50 (54%) procedures. The mean dose of administered doxorubicin was 26 ± 14 mg (9 – 60 mg) corresponding to 44% ± 23% (15–100%) of the entire dose.

### Response to cTACE and lipiodol retention pattern

Forty-six of the 82 HCCs (56%) were classified as CR, 7 (8%) as PR, 27 (33%) as SD and 2 (2%) as PD on first follow-up CT according to mRECIST.

Lipiodol retention was present in 72/82 (88%) of analyzed tumors. Lipiodol retention was considered to be complete in 30/72 (41%), and incomplete in 42/72 (58%) tumors. The remaining 10 (12%) tumors showed no lipiodol retention. The mean size of tumors showing complete, incomplete and no lipiodol retention was 23 ± 10 mm, 33 ± 16 mm, and 15 ± 5 mm, respectively (*p* < 0.01). Sixteen of the 46 nodules classified as CR (35% - mean 22.9 ± 8 mm) showed incomplete lipiodol retention, while 30 (65% - mean 22.8 ± 10 mm) showed complete lipiodol retention.

### Outcome of HCC showing CR on first follow-up CT

After a median follow-up of 14 months (3–35.9 months), tumor progression was observed a median of 12 months (3–31 months) after cTACE in 25/46 (54%) tumors with an initial CR. Median follow-up of tumors with persistent CR (i.e. no local progression) was 13.1 months (6 – 35.9 months). There were 15/16 (94%) and 10/30 (30%) tumors with incomplete and complete lipiodol retention that showed local progression on CT, respectively (*p* < 0.01), with no statistical difference in time to local progression (mean 11.1 ± 2 vs. 13.4 ± 3 months, respectively *p* = 0.51). Incomplete lipiodol deposition among nodules presenting with CR according to mRECIST had an OR of 30 (3.4 to 260 95%CI; p < 0.01) for further local progression. The characteristics of tumors with a CR according to lipiodol retention are set out in Table 2.

**Table 2:** Comparison of HCCs showing a complete response according to mRECIST with complete and incomplete lipiodol retention

	Incomplete (*N* = 16)	Complete (*N* = 30)	*p* value^+^
Serum AFP level (μg /L) ± SD	44 ± 110	93 ± 279	0.50
HCC Size (mm) ± SD	22.9 ± 0.8	22.8 ± 10	0.96
HCC location			1.00
- Left lobe	5 (32%)	10 (33%)	
- Right lobe	11 (68%)	20 (66%)	
Capsule appearance	4 (25%)	9 (30%)	0.50
Selective cTACE Treatment	10 (62%)	23 (76%)	0.24
Mean Delivered Dose (mg) ± SD	22 ± 11	26 ± 13	0.54
Embolic agent			
- Gelfoam	14 (87%)	26 (86%)	0.22
- PVA particles	0 (0%)	3 (10%)	
- None	2 (13%)	1 (4%)	
CBCT guidance	9 (56%)	17 (56%)	0.61
Local progression*	**15 (94%)**	**10 (30%)**	**<0.01**
Mean delay of local progression (months) ± SD	11.1 ± 2	13.4 ± 3	0.51

*median follow-up of 14 months (range 3.2–35.9 months)

+ Chi2 test and t-test were used for analysis of discrete and continuous variables respectively

*AFP* Alpha-fetoprotein, *CBCT* Cone beam computed tomography, *HCC* Hepatocellular carcionoma, *PVA* Polyvinyl alcohol, *SD* Standard deviation, *cTACE* Conventional transarterial chemoembolization

significant differences are bold

The mean delivered dose of doxorubicin was statistically lower in CR tumors with local progression than in those without local progression (35 ± 17 mg vs. 48 ± 23 mg, respectively; *p* = 0.03) (Table 3). There was no statistically significant difference in other factors including the appearance of imaging and the features of the cTACE technique between CR nodules with and without local progression (Table 3).

**Table 3:** Comparison of HCCs showing local progression and no local progression, in nodules classified with a complete response

	Local progression *N* = 25	No local progression *N* = 21	*p* value*
Serum AFP level (μg /L) ± SD	37 ± 18	122 ± 72	0.40
HCC size (mm) ± SD	23 ± 7	22.6 ± 12	0.78
HCC location			
- Right lobe	20 (80%)	14 (66%)	0.49
- Left Lobe	5 (20%)	7 (33%)	
Capsule Appearance	6 (24%)	7 (33%)	0.70
CBCT guidance	15 (60%)	10 (48%)	0.82
Selective cTACE Treatment	18 (72%)	15 (71%)	0.77
Mean Delivered Dose (mg) ± SD	35 ± 17	48 ± 23	0.03
Presence of gas on follow-up CT	1 (4%)	0 (0%)	0.93
Embolization Agent			
- Gelitaspon	23 (92%)	15 (71%)	0.11
- PVA particles	0 (0%)	3 (14%)	
- No embolization	2 (8%)	3 (14%)	

*Chi2 test and t-test were used for analysis of discrete and continuous variables respectively

*AFP* Alpha-fetoprotein, *CBCT* Cone beam computed tomography, *HCC* Hepatocellular carcionoma, *PVA* Polyvinyl alcohol, *SD* Standard deviation, *cTACE* Conventional transarterial chemoembolization

### Factors associated with local progression in CR tumors with complete lipiodol retention

The mean delivered dose of doxorubicin was statistically lower in tumors with local progression (mean 19 ± 12 mg vs. 29 ± 14 mg; *p* < 0.01). Other factors did not statistically differ between the groups. The mean size of tumors with a CR, complete lipiodol retention and local progression did not statistically differ from those without local progression (22 ± 9 mm vs. 23 ± 12 mm, respectively *p* = 0.78) (Table 4).

**Table 4:** Factors associated with local progression in HCCs showing a complete response according to mRECIST and complete lipiodol retention

	With local progression *N* = 10	Without Local progression *N* = 20	*p* value*
Serum AFP level (μg /L) ± SD	31 ± 36	124 ± 340	0.40
HCC size (mm) ± SD	22 ± 9	23 ± 12	0.78
HCC location			
- Right lobe	9 (90%)	14 (70%)	0.22
- Left Lobe	1 (10%)	6 (30%)	
Capsule Appearance	3 (30%)	7 (35%)	0.14
CBCT guidance	3 (30%)	10 (50%)	0.44
Selective cTACE Treatment	8 (80%)	15 (75%)	0.76
Mean Delivered Dose (mg) ± SD	19 ± 12	29 ± 14	<0.01
Presence of gas on follow-up CT	1 (10%)	0 (0%)	0.33
Embolization Agent			
- Gelitaspon	10 (100%)	16 (80%)	0.48
- PVA particles	0 (0%)	3 (15%)	
- No embolization	0 (0%)	1 (5%)	

*Chi2 test and t-test were used for analysis of discrete and continuous variables respectively

*AFP* Alpha-fetoprotein, *CBCT* Cone beam computed tomography, *HCC* Hepatocellular carcionoma, *PVA* Polyvinyl alcohol, *SD* Standard deviation, *cTACE* Conventional transarterial chemoembolization

## Discussion

In this study we showed that local progression occurred in almost all tumors presenting with both a CR according to mRECIST criteria and incomplete lipidodol retention on CT after a first session of cTACE, while this only occurred in one third of nodules with a CR and complete lipiodol retention.

Several authors have focused on the prognostic value of the lipiodol retention pattern after cTACE procedures [13, 16, 17]. The study by Takayasu et al. reported a good correlation between tumor necrosis on pathology and CT images when lipiodol retention was considered to represent necrosis [13]. Also, one study showed that a heterogeneous lipiodol pattern was correlated with a higher risk of recurrence [16], while another reported that the presence of lipiodol in at least 75% of the lesion was a predictor of improved patient survival [17]. Kim et al. reported better survival when compact lipiodol retention was observed in patients treated with TACE for unresectable HCC [18]. These results suggest that not only the presence but also the amount of lipiodol visualized on follow-up CT has prognostic value.

Nevertheless, certain authors have stated that value of the lipiodol retention pattern may not be fully accurate since the hyperattenuation of iodine retention may mask underlying viable parts of the tumor. It is important to note that from an oncological point of view, this is only challenging in tumors with a complete response, since patients with a partial response or stable disease – i.e. with persistent viable portions of treated tumors – need to be retreated whatever the lipiodol retention pattern. Interestingly, Dioguardi Burgio et al. have shown that tumors with a CR according to mRECIST and complete lipiodol retention had significantly higher rates of tumor necrosis on pathology than those with a CR but incomplete retention (mean 95% vs. 68%, respectively) [11]. Our results support these data, since local progression occurred in nearly all nodules with a CR and incomplete retention, while only one third in those with complete retention. This may have major consequences in clinical practice. Indeed, to date, a lesion with no residual arterial enhancement on CT is considered to be completely treated, whatever the type of lipiodol retention. These tumors are followed-up and treated when local progression is identified. The present results suggest that these tumors should be considered incompletely treated, and may benefit from a second session of TACE. This is particularly important in bridge therapy before liver transplantation [5, 19].

Incomplete lipiodol deposition appearance may be due to the incomplete catheterization and treatment injection in all tumor feeders. In this case, lesions typically show residual enhancement on follow-up CT. When all tumoral feeders are catheterized and treated, one could speculate that incomplete lipiodol deposition pattern could be related to the inability of lipiodol particles to penetrate the smallest tumor capillary vessels. Another explanation may be that partial lipiodol degradation may occur during the time interval between TACE and follow-up CT. Independently from of the mechanism, our results support the fact that incomplete deposition pattern should be considered as a marker of viable tumor, even in the absence of residual arterial hyper-enhancement.

Certain authors have suggested that MR imaging should be used instead of CT to assess tumor response after TACE. Hunt et al. [20] showed that the diagnostic accuracy of MR was better than CT (55% vs 43%) to assess tumor viability after TACE in a cohort of transplanted patients. Indeed, MR is considered to be more accurate than CT for the detection of tumoral remnants because its interpretation is not disturbed by lipiodol retention [12]. Moreover, accuracy can be increased by the use of image subtraction during arterial phase sequences [21].

Nevertheless, the level of evidence supporting these statements remains low, because the number of studies is limited and they include very small populations. Moreover, certain authors have reported a strong correlation between tumor devascularization on MR imaging and the amount of lipiodol retention on CT [22, 23]. However, MR imaging is not as accessible as CT, is more expensive, and patients may present with contraindications (i.e. pace makers or claustrophobia). Thus, CT is still routinely performed alone or in combination with MR imaging by many teams for patient follow-up after TACE treatment [6, 24, 25]. More importantly and besides these technical considerations, our results suggest that MR imaging may not be necessary in all patients, in particular in the absence of a CR in accurately performed CT, or in tumors with a CR and incomplete lipiodol retention suggesting the presence of remnant tumor cells, which may require retreatment. This suggests that an oncologically valid strategy would be to restrict MR imaging to patients with tumors showing both a CR and complete lipiodol retention on CT.

We could not identify any predictive factor for local progression except for the dose of chemotherapy delivered during treatment. While advantage of cTACE compared to arterial embolization alone is still a matter of debate for some [26, 27], this result supports the oncological value of chemotherapy agent injection, and suggests that the maximal possible dose should be delivered to each tumor target in order to reduce the risk of local progression. However, this factor should be considered with caution. Indeed, it was difficult to know the exact amount of chemotherapy delivered to a treated lesion because this depends on several elements, including treatment selectivity or the perfusion parameters of the tumor.

In addition to its retrospective design, our study has several limitations. First, the number of tumors showing a CR according to mRECIST and complete lipdiodol retention was fairly small. This can be explained by our inclusion criteria. Since we did not want to include previously treated tumors to avoid bias, we only included patients treated by a first session of cTACE. At the same time, the rate of tumors with a CR (56%) is similar to recent published series evaluating cTACE [6]. We were also not able to correlate local progression and lipiodol retention with tumor grading, because the diagnosis of most HCC was obtained by imaging criteria. Moreover, we did not focus on overall survival. Indeed, the goal of our study was to improve the understanding of the local reaction of tumors treated with cTACE, rather than to link lipiodol retention patterns to long term-outcome. Finally, we only included patients with up to three tumors. In case of multifocal HCC, it would have been impossible to confidently evaluate which nodules progressed.

## Conclusion

HCCs showing a CR according to mRECIST criteria on CT after a first session of cTACE should be divided into two groups with different potential local outcomes. Tumors with incomplete lipiodol retention have a high risk of local progression and should probably be retreated, and those with complete lipiodol retention with a much lower risk of local progression could probably benefit from MR imaging to identify viable tumor remnants.

## Data Availability

The datasets analysed during the current study available from the corresponding author on reasonable request.

## Abbreviations

CR: complete response
cTACE: conventional TACE
HCC: hepatocellular carcinoma

## References

[1] European Association for the Study of the Liver. Electronic address eee, European Association for the Study of the L (2018). EASL clinical practice guidelines: management of hepatocellular carcinoma. J Hepatol.

[2] Kim BK, Kim SU, Kim KA (2015). Complete response at first chemoembolization is still the most robust predictor for favorable outcome in hepatocellular carcinoma. J Hepatol.

[3] Gillmore R, Stuart S, Kirkwood A (2011). EASL and mRECIST responses are independent prognostic factors for survival in hepatocellular cancer patients treated with transarterial embolization. J Hepatol.

[4] Chapman WC, Majella Doyle MB, Stuart JE (2008). Outcomes of neoadjuvant transarterial chemoembolization to downstage hepatocellular carcinoma before liver transplantation. Ann Surg.

[5] Graziadei IW, Sandmueller H, Waldenberger P (2003). Chemoembolization followed by liver transplantation for hepatocellular carcinoma impedes tumor progression while on the waiting list and leads to excellent outcome. Liver Transpl.

[6] Bargellini I, Vignali C, Cioni R (2010). Hepatocellular carcinoma: CT for tumor response after transarterial chemoembolization in patients exceeding Milan criteria--selection parameter for liver transplantation. Radiology.

[7] Eisenhauer EA, Therasse P, Bogaerts J (2009). New response evaluation criteria in solid tumours: revised RECIST guideline (version 1.1). Eur J Cancer.

[8] Forner A, Ayuso C, Varela M (2009). Evaluation of tumor response after locoregional therapies in hepatocellular carcinoma: are response evaluation criteria in solid tumors reliable?. Cancer.

[9] Lencioni R, Llovet JM (2010). Modified RECIST (mRECIST) assessment for hepatocellular carcinoma. Semin Liver Dis.

[10] Bargellini I, Bozzi E, Campani D (2013). Modified RECIST to assess tumor response after transarterial chemoembolization of hepatocellular carcinoma: CT-pathologic correlation in 178 liver explants. Eur J Radiol.

[11] Dioguardi Burgio M, Ronot M, Bruno O (2016). Correlation of tumor response on computed tomography with pathological necrosis in hepatocellular carcinoma treated by chemoembolization before liver transplantation. Liver Transpl.

[12] Kloeckner R, Otto G, Biesterfeld S, Oberholzer K, Dueber C, Pitton MB (2010). MDCT versus MRI assessment of tumor response after transarterial chemoembolization for the treatment of hepatocellular carcinoma. Cardiovasc Intervent Radiol.

[13] Takayasu K, Arii S, Matsuo N (2000). Comparison of CT findings with resected specimens after chemoembolization with iodized oil for hepatocellular carcinoma. AJR Am J Roentgenol.

[14] de Baere T, Arai Y, Lencioni R (2016). Treatment of liver tumors with Lipiodol TACE: technical recommendations from experts opinion. Cardiovasc Intervent Radiol.

[15] Georgiades C, Geschwind JF, Harrison N (2012). Lack of response after initial chemoembolization for hepatocellular carcinoma: does it predict failure of subsequent treatment?. Radiology.

[16] Kinugasa H, Nouso K, Takeuchi Y (2012). Risk factors for recurrence after transarterial chemoembolization for early-stage hepatocellular carcinoma. J Gastroenterol.

[17] Vogl TJ, Trapp M, Schroeder H (2000). Transarterial chemoembolization for hepatocellular carcinoma: volumetric and morphologic CT criteria for assessment of prognosis and therapeutic success-results from a liver transplantation center. Radiology.

[18] Kim DY, Ryu HJ, Choi JY (2012). Radiological response predicts survival following transarterial chemoembolisation in patients with unresectable hepatocellular carcinoma. Aliment Pharmacol Ther.

[19] Millonig G, Graziadei IW, Freund MC (2007). Response to preoperative chemoembolization correlates with outcome after liver transplantation in patients with hepatocellular carcinoma. Liver Transpl.

[20] Hunt SJ, Yu W, Weintraub J, Prince MR, Kothary N (2009). Radiologic monitoring of hepatocellular carcinoma tumor viability after transhepatic arterial chemoembolization: estimating the accuracy of contrast-enhanced cross-sectional imaging with histopathologic correlation. J Vasc Interv Radiol.

[21] Kim S, Mannelli L, Hajdu CH (2010). Hepatocellular carcinoma: assessment of response to transarterial chemoembolization with image subtraction. J Magn Reson Imaging.

[22] Kwan SW, Fidelman N, Ma E, Kerlan RK, Yao FY (2012). Imaging predictors of the response to transarterial chemoembolization in patients with hepatocellular carcinoma: a radiological-pathological correlation. Liver Transpl.

[23] Shim JH, Han S, Shin YM (2013). Optimal measurement modality and method for evaluation of responses to transarterial chemoembolization of hepatocellular carcinoma based on enhancement criteria. J Vasc Interv Radiol.

[24] Schima Wolfgang (2007). Post-treatment imaging of liver tumours. Cancer Imaging.

[25] Yu JS (2005). Hepatocellular carcinoma after transcatheter arterial chemoembolization: difficulties on imaging follow-up. Korean J Radiol.

[26] Pleguezuelo M, Marelli L, Misseri M (2008). TACE versus TAE as therapy for hepatocellular carcinoma. Expert Rev Anticancer Ther.

[27] Facciorusso A, Bellanti F, Villani R (2017). Transarterial chemoembolization vs bland embolization in hepatocellular carcinoma: a meta-analysis of randomized trials. United European Gastroenterol J.

